# Utilization of Nanotechnology to Improve Bone Health in Osteoporosis Exploiting *Nigella sativa* and Its Active Constituent Thymoquinone

**DOI:** 10.3390/bioengineering9110631

**Published:** 2022-11-01

**Authors:** Javed Ahmad, Hassan A. Albarqi, Mohammad Zaki Ahmad, Mohamed A. A. Orabi, Shadab Md, Ritam Bandopadhyay, Faraha Ahmed, Mohammad Ahmed Khan, Javed Ahamad, Awanish Mishra

**Affiliations:** 1 Department of Pharmaceutics, College of Pharmacy, Najran University, Najran 11001, Saudi Arabia; 2 Department of Pharmacognosy, College of Pharmacy, Najran University, Najran 11001, Saudi Arabia; 3 Department of Pharmacognosy, Faculty of Pharmacy, Al-Azhar University, Assiut-Branch, Assiut 71524, Egypt; 4 Department of Pharmaceutics, Faculty of Pharmacy, King Abdulaziz University, Jeddah 21589, Saudi Arabia; 5 Department of Pharmacology, School of Pharmaceutical Sciences, Lovely Professional University, Phagwara 144411, Punjab, India; 6 Department of Pharmacology, School of Pharmaceutical Education and Research, Jamia Hamdard, Hamdard Nagar 110062, New Delhi, India; 7 Department of Pharmacognosy, Faculty of Pharmacy, Tishk International University, Erbil 44001, Iraq; 8 Department of Pharmacology and Toxicology, National Institute of Pharmaceutical Education and Research (NIPER)–Guwahati, Changsari, Kamrup 781101, Assam, India

**Keywords:** *Nigella sativa*, thymoquinone, nano-thymoquinone, antioxidant, anti-inflammatory, osteoporosis, nanotechnology-mediated drug delivery

## Abstract

Osteoporosis, a chronic bone disorder, is one of the leading causes of fracture and morbidity risk. Numerous medicinally important herbs have been evaluated for their efficacy in improving bone mass density in exhaustive preclinical and limited clinical studies. *Nigella sativa* L. has been used as local folk medicine, and traditional healers have used it to manage various ailments. Its reported beneficial effects include controlling bone and joint diseases. The present manuscript aimed to provide a sound discussion on the pharmacological evidence of *N. sativa* and its active constituent, thymoquinone, for its utility in the effective management of osteoporosis. *N. sativa* is reported to possess anti-IL-1 and anti-TNF-*α*-mediated anti-inflammatory effects, leading to positive effects on bone turnover markers, such as alkaline phosphatase and tartrate-resistant acid phosphatase. It is reported to stimulate bone regeneration by prompting osteoblast proliferation, ossification, and decreasing osteoclast cells. Thymoquinone from *N. sativa* has exhibited an antioxidant effect on bone tissue by reducing the FeNTA-induced oxidative stress. The present manuscript highlights phytochemistry, pharmacological effect, and the important mechanistic perspective of *N. sativa* and its active constituents for the management of osteoporosis. Further, it also provides sound discussion on the utilization of a nanotechnology-mediated drug delivery approach as a promising strategy to improve the therapeutic performance of *N. sativa* and its active constituent, thymoquinone, in the effective management of osteoporosis.

## 1. Introduction

Osteoporosis is a silent disorder causing reduced bone mass, altered microarchitecture of the bone, and contributing to low bone strength and high risk of fracture. It is estimated that 200 million women globally are affected by this disease, and approximately 8.9 million fractures occur annually due to osteoporosis [1]. The World Health Organization (WHO) defines osteoporosis through bone mineral density (BMD) evaluated by DEXA (dual energy X-ray absorptiometry) and reported as Z-scores and T-scores. The standard deviation of BMD that falls within 1 on this score scale is established as normal bone, the 1–2.5 score is classified as osteopenia (decreased bone mass), and more than 2.5 is considered osteoporosis [2].

**Figure 1:**
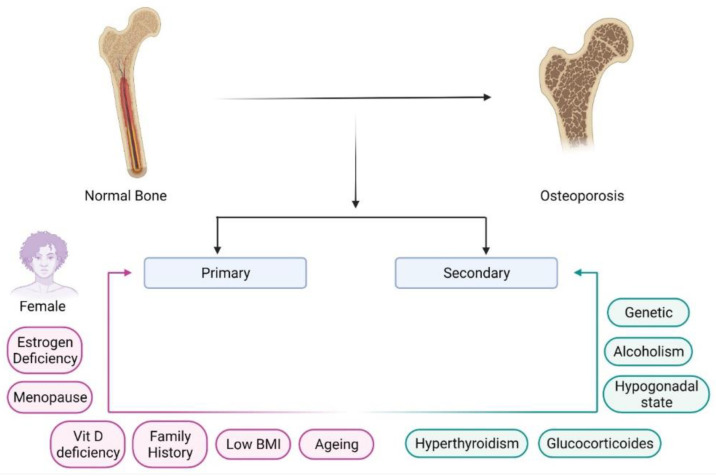
Primary and secondary causes of the development of osteoporosis. This image represents diverse causes of the transformation of normal bone towards osteoporosis. Primary causes include various factors within the body, and secondary causes have different pathological conditions. “Image created with BioRender.com”.

Osteoporosis has been classified as primary and secondary osteoporosis based on etiological factors (Figure 1). Primary osteoporosis may occur in both sexes but is more prevalent in postmenopausal women, while secondary osteoporosis results from pathological conditions such as hypogonadism or celiac diseases, and drugs such as glucocorticoids [3]. Osteoporotic fractures have many risk factors, including low peak bone mass, hormonal factors, use of steroidal medications, cigarette smoking, lack of physical activity, low intake of calcium and vitamin D, race, small body size, and a personal or family history of fracture [3,4]. In postmenopausal women, osteoporosis causes increased hip and vertebral fractures and a higher risk of falls. This further causes mortality and morbidity in postmenopausal women [5].

*Nigella sativa* (NS) is a dicotyledonous herb belonging to the Ranunculaceae family. NS seed is commonly recognized as black seed or black cumin. Numerous pharmacological activities, such as anticancer, anti-inflammatory, analgesic, antiasthmatic, antipyretics, antioxidant, antifungal, antimicrobial, and antiparasitic have been observed from the seeds and oil of NS [6,7]. NS is a hub of various chemical constituents, including essential oils, alkaloids, proteins, and saponins [6]. The biologically active components are α-hedrine, thymol, nigellicine, nigellone, nigellidine, carvacrol, thymohydroquinone, and thymoquinone (TQ) [8]. The active constituents also possess antihyperlipidaemic activity, wound healing activity, and protective activity for the cardiovascular, gastrointestinal, nervous, reproductive, and immune systems [9]. The significant properties of NS seed are also due to their richness in macro- and micro-minerals such as calcium, phosphorus, iron, magnesium, manganese, and copper. Mineralized bone is also composed of various trace elements that play an essential role in bone homeostasis [10].

The efficacy of NS has been evaluated for its pharmacological profile from its extract, oil, and its major active constituent, TQ. Over the years, TQ has been extensively studied for its effects on several diseases. Several preclinical and a few clinical studies have reported the effect of TQ in osteoporosis, but its impact on the disease is not significantly established in the existing literature. This review summarizes the current evidence from preclinical and clinical studies regarding the influence of NS and its active constituent, TQ, in osteoporosis. Furthermore, the manuscript discusses the significance of nanotechnology as a promising approach for delivering NS and its active component TQ in osteoporosis, which could be further helpful in improving its therapeutic performance.

## 2. Pathophysiology of Osteoporosis

Bone is an essential connective tissue in vertebrates. It is attached to the muscles, imparts structural support, shields vital organs, helps in locomotion and movement, provides a reservoir for minerals and growth hormones, and is a site for hematopoiesis [11]. Bone cells comprise mineral components that are organic (osteocalcin, type 1 collagen, and osteonectin) and inorganic (hydroxyapatite). Mature bone has two components (i) cortical bone, the outer hard and rigid bone matrix, and (ii) trabecular bone, which is an inner and spongy layer. The inner layer also acts as a site for bone remodeling [12]. Osseous cells undergo modeling and remodeling during the life span, allowing bone cells to grow radially and longitudinally. Bone modeling occurs during childhood to alter the shape and adjust the skeleton due to physiological and mechanical pressures.

In comparison, maintenance of mineral homeostasis for bone strength occurs during bone remodeling, which continues for life [13]. During a lifetime, the skeleton undergoes many modifications through a process known as bone remodeling. The process involves a steady state of bone resorption, new bone formation, healing of the fracture, and bone strengthening. The bone remodeling process involves different stages. Initially, osteocytes of quiescent bone detect physiological, mechanical, and hormonal signals and activate the osteoclast cells. Activated osteoclast cells attach to the bone surface, degrading bone matrix with the enzyme cathepsin K and a decrease in pH. This initiates the bone resorption process. Further, osteoclast cells undergo apoptosis to end the bone resorption process. In contrast, activation of osteoblast cells releases the enzyme alkaline phosphatase, osteocalcin, and collagen synthase, which improve the mineralization of bone and enhance bone density. Homeostasis is regulated between the osteoclast and osteoblast cells to maintain normal bone health.

Osteoid is produced, leading to the maturity of bone matrix and subsequently causing its mineralization. The proteins secreted by osteoblasts heal the cavities formed during matrix degeneration. The final stage is the termination phase, in which bone mineralization occurs until the new bone is completely replaced with the old bone [14]. Remodeling of bone takes weeks. The balance between bone formation and resorption is seen in healthy individuals as their bone matrix is dense and strengthened. Collagen provides elasticity and flexibility, whereas calcium and phosphate provide strength and rigidity. However, in osteoporosis, the porosity of tubercular bone increases, and a reduction in bone density is observed [15].

Several factors are attributed to the pathology of osteoporosis, responsible for compromised strength and fragility of the skeleton. These factors include inadequate bone microarchitecture, defects in bone tissue, insufficient repair of microdamage due to stress, and higher bone resorption during bone remodeling [16]. However, as women age, there is increased resorption, causing an imbalance in bone formation and reduced bone mass. Bone formation is mediated by osteoblast cells; bone resorption is mediated by the activity of osteoclast cells along with certain chemicals and hormones such as estrogen, calcitonin, parathyroid hormone, vitamin D, and calcium [17]. Osteoclasts are the cells derived from hematopoietic stem cells, which function to reabsorb bone.

In contrast, osteoblasts cells are derived from mesenchymal stem cells, and they work with the help of supporting tissues such as blood vessels, nerves, and connective tissue [14]. An imbalance between bone resorption and bone formation leads to abnormal bone remodeling, causing bone disorders such as osteoporosis [18]. The spongy or trabecular part (20% of bone) begins to decline with age. In contrast, dense/outer/cortical bone begins to fall in the elderly, due to menopause and decreased estrogen levels, leading to reduced bone mineral density [17].

## 3. Phytochemistry of *N. sativa*

**Figure 2:**
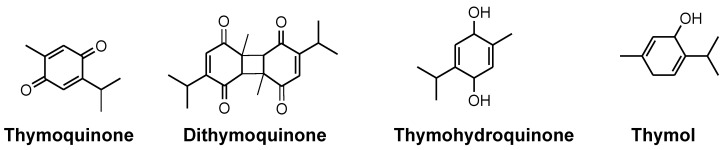
Chemical structures of major constituents of *N. sativa*.

NS is a dicotyledonous plant belonging to the Ranunculaceae family. It is also known as “crowfoot” or “buttercup”. Ranunculus is the largest genera from the family of more than 2000 flowering plants, with 43 genera widely distributed around the globe [19]. Reports about the chemical constituents of NS suggested the presence of oils, carbohydrates, proteins, and fibres [20]. Several experimental studies have suggested the medicinal importance of NS, which is primarily due to the presence of quinone constituents (TQ) [21,22]. Large amounts of carbohydrates, proteins, fats, vitamins, minerals, and fiber make the seed nutrition rich. Several amino acids have also been detected in the plant, such as aspartate, glutamate, arginine, methionine, and cystine. Vitamins identified in black seed include folic acid, niacin, thiamine, and pyridoxine. Minerals such as calcium, iron, phosphorus, zinc, and copper are also found in it. A phytochemical investigation of black seeds revealed the presence of sterols, alkaloids, essential oil, and saponins. The seeds of this herb contain palmitic acid (20.4%) and linoleic acid (64.6%) as primary fatty acids in a fixed oil (26–34%). From the nutrient pool of NS, the bioactive compound TQ has been identified as possessing significant medicinal potential. Extensive research has been conducted, and is ongoing, in order to establish and validate the benefits of NS and its constituents, as claimed historically [23]. Apart from TQ, NS also contains limonene, thymol, *p*-cymene, carvacrol, 4-terpineol, *α*-pinene, longifolene, and *t*-anethole benzene, thymohydroquinone, dihydrothymoquinone, carvacrol, thymol, *α*-thujene, *α*-pinene, *β*-pinene, *t*-anethole, and γ-terpinene as volatile principles [19]. Some of the important constituents of NS have been illustrated in Figure 2.

## 4. Pharmacology of *N. sativa*

**Figure 3:**
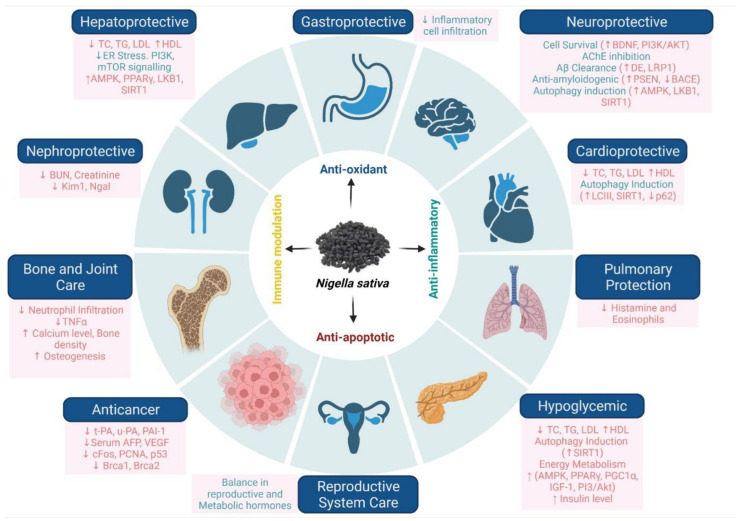
Detailed pharmacological profile of *N. sativa* and summary of its protective mechanisms in various organ-based diseases. Up arrow (↑) indicates increase in level and down arrow (↓) indicates decrease in level. “Image created with BioRender.com”.

TQ is an important major phytoconstituent of NS and its volatile oil. TQ stimulates a variety of pharmacological effects, including hepatoprotective [24,25], nephroprotective [26,27], antimicrobial [28], anti-inflammatory [29,30], antioxidant [30], antifungal [31], anticancer [32,33,34], anti-Alzheimer [35,36,37], anti-Parkinson [38,39,40], anticonvulsant [41,42], antidepression [43,44], and many more (illustrated in Figure 3).

Numerous In vitro and in vivo studies have demonstrated the therapeutic benefits of TQ and black cumin [45,46]. It has exhibited its potential as an antimicrobial, anticancer, anti-inflammatory, antifertility, antioxidant, analgesic, and antipyretic agent. It has also proven beneficial for patients with diabetes, hypertension, metabolic syndrome, asthma, dyslipidemia, and convulsions. It has a broad spectrum of antimicrobial effects on various pathogens such as fungi, bacteria, viruses, parasites, and Schistosoma. In different in vivo animal models, TQ has shown efficacy towards many cancers, such as fibrosarcoma, breast, pancreas, blood, lung cervix, prostate, and colon cancer. Studies have also suggested that certain diseases of the kidney and liver, in addition to neurobehavioral disorders, can be prevented with optimum utilization of black cumin and TQ. Results from various clinical trials also exhibited therapeutic benefits of TQ on numerous diseases of the skeletal system, cardiovascular system, respiratory system, nervous system, gastrointestinal system, and reproductive system, as well as skin diseases [19,47].

## 5. Protective Mechanisms of *N. sativa* and Its Active Constituents against Osteoporosis

Loss of bone mass in osteoporosis is generally encountered in the trabecular region of the bone. Downregulation in bone biochemical markers, such as osteocalcin—the marker of bone formation, elevation in cross-linked C-telopeptide, and bone resorption are associated with osteoporosis [7,48]. Menopause can also be a reason for osteoporosis, due to estrogen deficiency, which induces bone resorption.

Several extracts of other medicinal plants such as soy, blueberry, *Achyranthes bidentata*, and *Labisia pumila* have been examined in postmenopausal osteoporotic animal models [49,50,51,52]. However, NS has not been evaluated in animal models of postmenopausal osteoporosis. One clinical trial has reported no activity of NS, but the results of that experiment are not reliable due to the short treatment period, noncompliant formulation, and small population size [53].

Diabetes is another crucial, worldwide-accepted reason for osteoporosis. Although the exact mechanism is unknown, diabetes is hypothesized to induce osteoporosis-like conditions due to insulin deficiency, insulin resistance, hyperglycemia, or atherosclerosis [54]. Anabolic effects of insulin and insulin-like growth factors can also possibly contribute to diabetes-induced bone deformities [55]. In two streptozotocin-induced rat diabetic models, it was observed that NS treatment was able to alleviate the diabetics-induced bone changes [45,56]. The mechanism of its action is still not precisely understood, but it was hypothesized that metabolic changes due to an improvement in the blood sugar level is the primary reason for its bone-protective effects. In a few parts of the world, NS has even been exploited for its antidiabetic properties [57]. NS is also reported to reduce hyperglycemia in streptozotocin-induced diabetic rat models, upregulation of serum insulin expression, regeneration of pancreatic beta cells, and increased insulin in immunoreactive *β*-cells [58,59,60,61,62]. Thus, further clinical evaluation of NS has the potential to provide treatment alternatives to traditional antidiabetic and antiosteoporosis drugs.

### 5.1. Antioxidant Activity of N. sativa in Osteoporosis

Patients with osteoporosis experience oxidative stress as their lipid peroxidation levels and antioxidant enzyme levels are downregulated. This makes hypertensive, diabetic, and smoking-addicted patients more susceptible to osteoporosis [26,27,28,29,30]. In these patients, exposure to oxidative stress can result in bone mineral density reduction and, ultimately, osteoporosis. In studies, ferric nitrilo-triacetate (FeNTA) was used to induce bone density loss via the production of reactive oxygen species which, with the help of ferric ions (Fe^3+^), ultimately increased the oxidative stress inside the cells [63,64]. This induces bone damage by inducing lipid peroxidation inside the bone cells [65,66]. This can lead to stimulated osteoclast formation, impaired osteoblastic function, decreased recruitment of osteoblasts, and impaired collagen synthesis [63,64,65,66,67]. The generated free radicals can also activate NF-kB and upregulate bone-resorbing cytokines IL-6 and IL-1, thus increasing the oxidative stress inside the cells [63,68].

Therefore, it appears that oxidative stress leads to bone density reduction, and antioxidants can alleviate oxidative stress-mediated osteoporosis by blocking the harmful effects of reactive oxygen species [63]. It was observed that potent antioxidant agents such as tocotrienol and tocopherol inhibit the activities of FeNTA, downregulate oxidative stress, and inhibit osteoporosis [63].

TQ is the most abundant constituent of NS extract. It was observed that the most significant activity of TQ is the antioxidant effect via free radical scavenging, which was found to be equipotent to superoxide dismutase enzyme [69]. As an antioxidant, TQ’s main action is superoxide scavenging, thus inhibiting the activation of osteoclasts [70]. In a cancer model, TQ was observed to reduce FeNTA-induced oxidative stress, hyperproliferative response, and renal carcinogenesis [71]. In rheumatoid arthritis (RA) models, TQ reduced the expression of IL-1, TNF-α, bone turnover markers, alkaline phosphatase, and tartrate-resistant acid phosphatase, which indicates the proper balance in the bone sorption and resorption activities. In addition, TQ was found to downregulate NF-kB activation in a time-dependent manner [72]. Thus, it can be concluded that the antioxidant activities of NS can be very beneficial in the management of osteoporosis.

### 5.2. Anti-Inflammatory Activity of N. sativa in Osteoporosis

**Figure 4:**
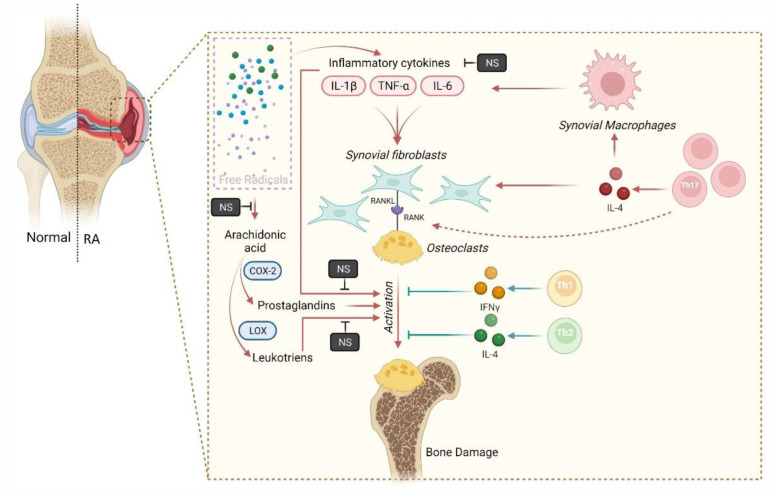
Schematic representation of protective mechanisms of *N. sativa* against osteoporosis associated with RA. Stromal stem cells and osteoblasts express receptor activators of NF-κB ligand (RANKL). The binding of RANKL to its receptor, RANK, on the surface of osteoclasts and their precursors regulates the differentiation of precursors into multinucleated osteoclasts. Further osteoclast activation increases bone resorption. Stromal stem cells and osteoblasts also secrete osteoprotegerin (OPG), which binds with RANKL and prevents its interaction with RANK, thus protecting from excessive bone resorption processes. The RANKL/OPG ratio is an essential determinant of bone mass index in pathological conditions. TQ is known to prevent RANKL-induced osteoclastogenesis activation and osteolysis in an experimental inflammation model. “Image created with BioRender.com”.

Anti-inflammatory activity can prove to be very effective in reducing osteoporosis, as experimental findings suggest a direct link between inflammation and osteoporosis [73,74]. A higher incidence of osteoporosis was associated with inflammatory conditions such as ankylosing spondylitis, RA, and systemic lupus erythematosus [75,76,77,78]. There is a negative correlation between C-reactive protein, a marker of systemic inflammation, and bone density, suggesting inflammation’s role in reducing bone density, i.e., osteoporosis. Interestingly, it was noticed that systemic inflammations result in general bone loss, whereas local bone loss is restricted to only the region of local inflammation [73]. Upregulation of age-driven proinflammatory cytokines, gouty arthritis, RA, and psoriatic arthritis can also exacerbate osteoporosis progression [79,80,81,82,83,84]. The consolidated mechanistic perspective of *N. sativa* against osteoporosis associated with RA is illustrated in Figure 4.

Inflammation is generally mediated by two enzymes, cyclooxygenase (COX) and lipoxygenase (LOX). They then activate the downstream inflammation pathways by generating prostaglandins (PGs) and leukotrienes (LTs). Thus, PGs and LTs are the main mediators of inflammation [84,85]. In rat peritoneal leukocyte cells where inflammation was induced with calcium ionophore A23187, TQ inhibits the generation of LTs and PGs via inhibiting COX and LOX in a dose-dependent manner [86,87,88].

Indomethacin is an established anti-inflammatory drug. TQ was found to be equipotent to indomethacin in inhibiting COX activity in an In vitro investigation of COX assay. Therefore, TQ or NS extracts can also be studied as potential alternatives to NSAIDs in reducing inflammation [89]. As inflammation is directly linked to osteoporosis, NS can also be used as a potential antiosteoporosis agent. Another possible anti-inflammatory mechanism of TQ is the interruption of NO fabrication by macrophages [90]. Oral administration of NS was also observed to reduce carrageen and formalin-induced paw edema, suggesting its possible anti-inflammatory activity [91,92].

In rats, the inflammation of ligaments and bones connecting teeth in periodontitis was reduced by intragastric administration of TQ. A reduction in osteoclast number and increased osteoblastic activity were noticed after TQ administration. Due to this, these rats encountered alveolar bone loss due to periodontitis. TQ administration reduces alveolar bone loss via acting as an anti-inflammatory and antioxidative agent [93].

### 5.3. Bone Regeneration Activity of N. sativa

NS extracts have shown good bone formation and healing in various osteoporotic models [94]. Regenerated bones after NS treatment have shown detailed blood vessels in the bone marrow. The tooth sockets after recovery have also shown dense trabeculae and large aggregations of astrocytes [95]. NS treatment was also observed to prompt osteoblast proliferation, ossification, and decreasing osteoclasts in ovariectomized osteoporotic animals [10]. Oral administration of TQ could also reverse bone defects in rats, but further investigation using different doses and local routes of administration is recommended [96]. Incubation of the third molar teeth of human subjects (15–20 years of age, n = 5) results in clear and compact calcium granules. Thus, it represents clear osteogenic differentiation [97]. A clinical study has been performed to evaluate the bone regeneration power of NS extract. Improvement in bone quality, i.e., increased bone density and gain in peri-implant tissue, was reported after 3 to 6 months [98]. Thus, it can be concluded that NS extracts can potentially treat bone deformities; however, more studies are needed to convert the preclinical finding into clinically significant results.

## 6. Palliative Behavior of *N. sativa* in Osteoporosis

The seed of NS has been researched and utilized pharmacologically for its extract, oil, and its major active constituents, such as TQ. We summarize the pharmacological benefits of various components of NS studied in various preclinical and clinical investigations, which provide evidence for its utilization in the effective management of osteoporosis.

### 6.1. N. sativa Seed Extract

A research study emphasized the effect of NS seed powder (800 mg/kg) in the treatment of osteoporosis by using 30 ovariectomized female Wistar rats as postmenopausal osteoporosis models. The study was conducted for 12 weeks. NS powder was dissolved in distilled water and administered to animals through gavage. The result showed that, in the NS treated group of animals, the NS effectively improved the decreased levels of calcium, and animals treated with the NS exhibited a significant rise in suppressed levels of bone formation markers, such as bone alkaline phosphatase, malondialdehyde, amino-terminal collagen type-1 telopeptide, TNF-*α*, nitrates, and IL-6. Histological examination of the tibia bone in the NS group showed an enhancement in the thickness of trabecular and cortical bones. Further, histological investigation of the liver showed higher levels of unsaturated fatty acids, demonstrating the herbal extract’s anti-inflammatory and antioxidant potential. Additionally, it was found that without mononuclear infiltration of cells, the blood vessels were comparatively relaxed at the portal site [99].

Research with animal models examined the bone healing potential of NS extract on calvarial defects. NS seed extract (10 mg/kg/day) was administered (to 32 female Wistar rats) with a trephine burr on each calvarium for 12 weeks. Investigation regarding the presence of inflammation, bone metabolism, and the presence of the number of bone cells (osteoblasts and osteoclasts) was performed. The outcome did not show any inflammation. There was mineralization observed at the calvarial-defective site. Increased osteoblastic activity and enhanced mineralization were also indicative of ossification, as observed in histomorphometry analysis, indicating the bone healing potential of NS [10].

Another research study elucidated the beneficial effects of NS extract on an OVX rat model. The researchers administered various doses of NS extract (5, 10, and 20 mg/kg/day) orally for 8 weeks to female OVX rats. The thickness of the bone matrix was assessed by staining them with hematoxylin dyes and eosin. Flow cytometry was employed to identify the concentrations of bone resorption genes TRAF6 (tumor necrosis factor receptor-associated factor) and NAFTc1 (nuclear factor of activated T-cells cytoplasmic1). The results showed that NS positively improved osteoporosis through significant suppression of TRAF6 and NAFTc1. An amount of 10 mg/kg of NS extract showed maximum efficacy [100].

In a rare model of osteoporosis, ovariectomized female Wistar rats were subjected to plaster casts in the left hind legs to promote osteoporosis. Dry bone weight was measured to identify bone loss in an animal model. There was higher bone weight gain after administration of NS extract, independent of its concentration. NS (800 mg/kg) treatment in combination with raloxifene (5.4 mg/kg) significantly increases bone mass and calcium levels compared to individual therapy. Further, histological evaluation and radiographic investigation showed that NS extract increased the activity of osteoblasts and suppressed osteoclastic function, suggesting the potential of NS in the bone formation process and, thus, in preventing osteoporosis [101].

A study showed that, when supplemented with food, *N. sativa* (1%) could elevate parathyroid hormone levels in adult male diabetic rats. When it was combined with nano-sized clinoptilolite, it significantly boosted the bone marker osteocalcin and enhanced bone mineralization in an animal model [102].

### 6.2. N. sativa Oil

A group of scientists studied the therapy of seed extract of NS, as oil (2 mL/kg/d i.p) alone and in combination with human parathyroid hormone (hPTH) (6 µg/kg/day i.p.), for its effect on bone mass and the mechanical properties of bone and bone microarchitecture in streptozotocin-induced diabetic insulin-dependent Wistar albino rats during 4 week therapy. The results showed that NS treatment (singularly and in combination with hPTH) causes a decrease (˂0.05) in serum glucose and an increase (˂0.001) in serum insulin levels in streptozotocin-induced diabetic rats. NS treatment (singularly and in combination with hPTH) remarkably enhanced the environment of insulin immunoreactive *β*-cells. The research claimed that NS’s safety and efficacy are comparable to insulin therapy. The effect of NS was also corroborated by the effects of insulin in improving diabetic osteopenia. The treatment with NS in combination with hPTH significantly improved bone mass, trabecular connectivity, and mechanical strength compared with single therapy with NS and hPTH in insulin-dependent diabetic rats [45].

The same research group evaluated the effect on skeleton assessed with bending analysis for mechanical strength using the finite element method. The concentrated force was applied at midpoint through the bone’s length to determine bone displacement and strain. The outcome showed that the combined effect of NS (seed extract) and hPTH were more efficacious in animal models identified through mechanical strength and bone histomorphometry of femur and vertebrae [46].

A pilot study was executed by Valizadeh et al. for 3 months in 15 postmenopausal osteoporotic women of the age group 48–74 years for 12 weeks. The study aimed to investigate the anabolic influence of black seed oil on bone metabolism through the assessment of biochemical markers of bone (bone alkaline phosphatase, carboxy-terminal crosslinked telopeptide, and osteocalcin). GC-MS analyzed NS oil in the study, and it demonstrated the presence of *o*-cymene (30.5%), TQ (22%), and *α*-thujene (14.7%) as major constituents. The study outcome revealed that NS oil (0.05 mL/kg/d) did not improve bone turnover. It also did not improve the bone mineral density of the total hip and lumbar spinal region. However, investigators suggested that higher tolerable doses with a larger sample size may be employed for further studies to obtain better clarity for its therapeutic use in osteoporosis [103].

To elucidate the protective role of NS oil, Mohammad et al. ran a comparative study of NS oil (800 mg/kg) with estradiol (0.1 mg/kg) and alendronate (0.1 mg/kg) in prednisone-induced osteoporosis in a female albino rat model. The drugs were administered orally, alone and in combination, for 6 weeks, and the results were assessed using bone markers. The results showed that NS oil alone significantly reduced urinary hydroxyproline and elevated levels of serum calcium, bone alkaline phosphatase, and osteocalcin. NS oil and alendronate combination also exhibited similar results. These results demonstrated enhanced bone formation and suppressed bone resorption with NS oil alone and combined with alendronate [104].

In an In vitro study on C2C12 cell lines, NS was combined with hydroxyapatite (HAP) to establish an orthopedic bone scaffold. This was performed to study the bone regeneration capacity of both components. The NS-coated HAP scaffold is also economical. Scanning electron microscopy, Fourier-transform infrared spectroscopy, and X-ray crystallography were used to analyze the scaffolds. The results showed that NS-grafted HAP scaffolds possess good potential for regenerating skeletal muscles, as they enhanced the differentiation of osteoblasts and myoblasts. The biocompatibility of the NS-coated HAP scaffold was tested in In vitro C2C12 myoblast cell lines, and the study shows a significant growth in cells, with no negative impact. NS oil-coated HAP scaffolds were also tested for inhibition of *Staphylococcus aureus* biofilm. The scaffolds demonstrated an antibacterial effect against the *S. aureus* strain [105].

Another study was conducted on 120 postmenopausal women with decreased bone density who were aged between 50–65 years. The combined effect of NS oil (one capsule of 1000 mg/day) with nano-micelle curcumin (one capsule of 80 mg/day) was studied in a triple-blind randomized, placebo-controlled clinical trial for 12 months. Apart from age, patients also had similarities in other baseline characteristics such as weight, calorie intake, exercise, physical activity, bone mineral density, history of breastfeeding, contraception, and menopause (past years). The combination of NS oil with nano-micelle curcumin produced minimal side effects such as nausea, vomiting, belching, headache, and unpleasant taste. The impact of NS on the expression of various genes such as miRNA-503, miRNA-21, and miRNA-422a was also evaluated. These genes play an essential function in life processes of inflammation, production, growth, migration, differentiation, and cell death. The outcome of the trial demonstrated that the combination of NS oil with nano-micelle curcumin elevated the plasma levels of miRNA-21 in subjects with low bone mineral density. This indicates the positive role of NS oil with nano-micelle curcumin in bone homeostasis. The researchers also suggest future studies with a bigger sample size to establish the effect of NS on bone biology [106].

### 6.3. Thymoquinone (TQ)

In an In vitro study, TQ was assessed for cell proliferation using MC3T3-E1 cell lines (an osteoblast precursor cell line). TQ at a higher concentration (10–40 µM) significantly induced the growth of MC3T3-E1 cells at 24, 48, and 72 h incubation. The authors further cultured MC3T3-E1 cells for 14 days under mineralizing conditions with TQ (1, 5, 10, and 20 μM), and In vitro mineralization was measured by calcium deposition using Alizarin Red staining. The study shows that TQ treatment significantly causes mineralization, proliferation, and differentiation in murine bone cell lines. The bone formation markers osteocalcin and alkaline phosphatase were elevated, as were osteopontin levels. The effects of TQ are also attributed to the enhancement of signaling pathways by extracellular signal-related kinase genes (ERK), which increased expressions of bone morphogenic proteins (BMP-2). The study endorsed the anabolic effect of TQ on the skeleton, benefiting osteoporosis [107].

In another In vitro study, TQ was studied on RAW 264.7 (murine macrophage) and MC3T3E1 (murine preosteoblasts) cell lines. TQ in different concentrations (2.5, 5, and 7 µM) exhibited promising results. It showed suppression of signaling of NF-κB and mitogen-activated protein kinase (MAPK), consequently inhibiting RANKL-induced osteoclastogenesis. The signaling mechanism was suppressed as TQ interfered with the phosphorylation of IkB kinase. Further, TQ also downregulated the expressions of osteoclastic markers such as TRAP, c-FOS, NFATc1, and DC-STAMP. Additionally, TQ revealed its antioxidant activity in these cell lines as it reduced the generation of reactive oxygen species induced by RANKL, aiding improvement in osteoporosis [108].

TQ was loaded in the combination of hydroxyapatite and alginate scaffolds at a concentration of 25 µM and 50 µM to evaluate its osteogenic potential in human adipose-derived mesenchymal stem cells (hADMSCs). It was observed that these doses were nontoxic for hADMSCs. The study concluded that after 14 days of seeding, the combination enhanced the capability of TQ to upregulate osteoblastic differentiation in hADMSCs In vitro. Additionally, in the presence of an osteogenic medium for 28 days, TQ was shown to enhance the expressions of bone turnover markers bone alkaline phosphatase, osteocalcin, collagen 1, and osteopontin. The study showed the synergistic action of TQ and hydroxyapatite to accelerate and boost the osteogenic potential of bone marrow-derived mesenchymal stem cells (BM-MSCs). The study also supports the positive role of TQ in bone metabolism [109]. Table 1 summarizes the pharmacological benefits of different components of NS (seed extract and seed oil) studied in various In vitro cell lines and preclinical investigations in the effective management of osteoporosis.

As evidenced through studies, the NS seed, its oil, and the active constituent TQ have been investigated for their antiosteoporotic potential [110]. Existing research emphasizes NS’s capacity to improve bone formation by targeting bone formation markers, especially BALP (bone-specific alkaline phosphatase) and osteocalcin, among others. NS increases bone mineralization, thus improving bone ossification. The benefits of NS in osteoporosis showed gene-related attenuation of inflammation, increased BMP-2 activity, elevated osteoblastic activity, and suppressed osteoclastogenesis. However, to substantiate these effects for precise and long-term human use, more clinical trials need to be designed to establish the osteoblastogenesis actions of NS. Clinical trials involving both genders and a larger number of volunteers of varying age groups may support the research most. The impact on specific bone formation markers needs to be analyzed as a secondary outcome in clinical studies.

## 7. Nanotechnology-Mediated Bone-Specific Delivery of NS and/or TQ in Osteoporosis

Compared to semisynthetic and synthetic drugs, medicinal plants are easily accessible, have lesser side effects, and are economical. Moreover, medicinal plants and herbs have traditional values in various cultures and religions. NS has been employed as a remedy for numerous infections (fungal, bacterial, viral, and parasitic), and chronic diseases (cardiovascular, neurological, cancer, diabetes, etc.) for many years [23]. The published researches corroborate the beneficial pharmacological profile of NS seeds, oils, and principal constituents [110]. TQ, the most important phytochemical of this plant, has potency as an immunomodulatory, antioxidant, antimicrobial, anti-inflammatory, and anticancer agent. It has also exhibited its efficacy as a nephroprotective, neuroprotective, antidiabetic, hypolipidemic, and antihistaminic, along with benefits in reproductive and joint diseases [8]. In addition, the ancient herb NS exhibits promising protection from bone deterioration during osteoporosis. However, there is not enough evidence regarding its benefits in bone homeostasis [10]. The clinical utility of this classic herb is still being scrutinized although, over the years, several pieces of research have revealed the medicinal advantages of TQ and black seeds. TQ exhibits minimal or no toxicity, but its low bioavailability is a significant hindrance to its therapeutic application in various communicable/noncommunicable diseases. The main reason behind its low bioavailability is poor aqueous solubility, low stability at biological pH, and high sensitivity to light exposure even for a short period. It is widely reported that TQ is highly unstable in an aqueous medium at prominent light and pH [111]. Nanotechnology provides an opportunity to overcome the biopharmaceutical challenges in its drug delivery approach for enhancing therapeutic efficacy in various disease conditions.

### Nanotechnology-Mediated Drug Delivery

**Figure 5:**
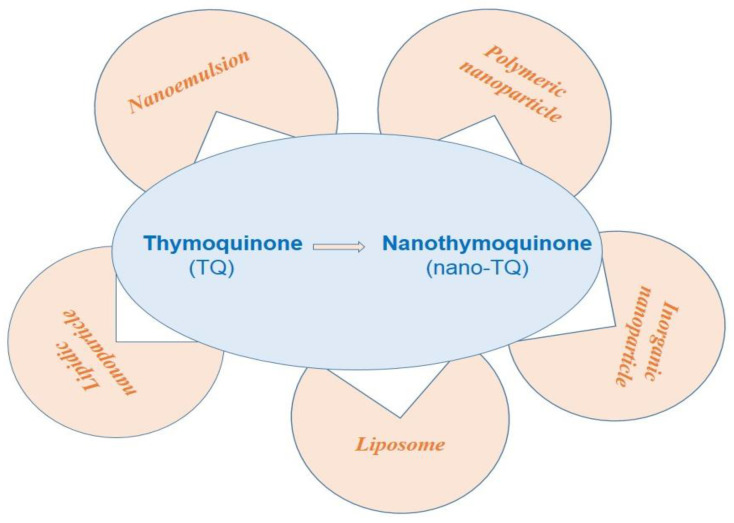
Various nanotechnology approaches improve the biopharmaceutical characteristics of TQ in the form of nano-TQ.

Nanotechnology is a promising approach to improving the biopharmaceutical performance of low bioavailable, bioactive molecules of plant origin [112]. The therapeutic efficacy of TQ encapsulated inside various nanoformulation systems (illustrated in Figure 5) as nano-TQ resulted in a significant increase in intended pharmacological activity compared to the conventional TQ formulation system [113].

The biopharmaceutical characteristics of nano-TQ (such as aqueous solubility, stability, and permeability across the biological membrane) have been greatly improved compared to TQ as bioactive therapeutics [114,115] that are ultimately responsible for the increase in therapeutic efficacy in disease conditions of various communicable/noncommunicable disorders (summarized in Table 2).

**Figure 6:**
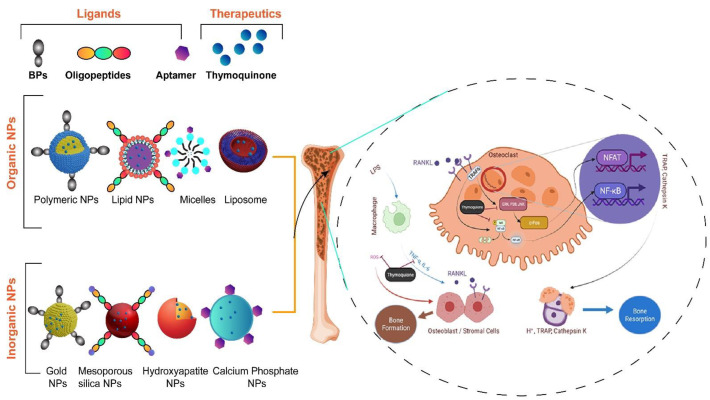
The illustration highlights bone-specific targeted delivery of NS/TQ utilizing the ligands-conjugated nanoparticulate system to improve the therapeutic performance of loaded bioactives (NS/TQ) in osteoporosis. Ligand-conjugated nanoparticulate systems of organic or inorganic origin bind to the target sites of bone tissue for site-specific delivery of loaded therapeutics (NS/TQ) in bone cells (osteoblast, MSCs, and osteoclast) to promote bone formation and inhibit bone resorption. “Image created with BioRender.com”.

Nanotechnology is utilized as a promising approach for bone-specific drug delivery, which may include organic and inorganic nanoparticles (NPs). The example of NPs of organic nature may consist of polymeric NPs, lipidic NPs, micelles, liposomes, etc. In contrast, NPs of an inorganic nature may consist of metallic NPs, mesoporous silica NPs, hydroxyapatite NPs, calcium phosphate NPs, etc. Different types of targeting ligands (such as bisphosphonates (BPs), oligopeptides, aptamers, etc.) are conjugated with the nanoparticulate system for bone-specific targeted delivery, ultimately further helping to improve the efficacy of loaded therapeutics (pharmaceuticals and/or phytopharmaceuticals) to alleviate the side effects of conventional therapy [125]. The bone-specific targeted delivery of NS components and its active constituent TQ in exploiting a ligands-conjugated nanoparticulate system could be promising for the better management of osteoporosis (illustrated in Figure 6).

Evidence shows that major studies on NS and its active constituent TQ are conducted on cell lines and preclinical models [126,127]. Hence, to establish and validate the biopharmaceutical attributes, pharmacology, and toxicology of TQ/nano-TQ, extensive clinical investigations are required for its further utility in the effective management of osteoporosis [19,26].

## 8. Conclusions and Future Directions

NS and its active constituent TQ exhibit good safety and efficacy for numerous diseases. Therefore, to realize its capacity in bone disorders, it needs to be assessed through clinical studies. There is limited data regarding clinical studies on the effect of black seeds on osteoporosis therapy. Initially, the outcome of the research was not sufficient to be acknowledged. Some studies even suggested that the chemical constituent from the herb is not effective for use in treatment of bone disorders such as osteoporosis. The existing treatment of osteoporosis is expensive, has many side effects, and does not provide satisfactory recovery. TQ may serve as a potential alternative. This review provides future directions to attract more research to target and focus on the bone-healing properties of this seed, its active constituents, and their positive impact on the functioning of osteoblasts and bone formation. It would aid researchers in identifying and investigating different osteoporosis models for future preclinical and clinical studies on NS, TQ, and other constituents of NS. However, employing a larger sample for a longer duration is suggested to authenticate and establish the results obtained from clinical studies.

## Figures and Tables

**Table 1:** Summary of the pharmacological profile of *N. sativa* and its constituent TQ, promising in the management of osteoporosis.

S. No.	Part of NS	Experimental Model	Molecular Mechanisms/Significant Outcome	Ref.
1.	Seed volatile oil (100 mg/kg, i.p.) for 4 weeks	Streptozotocin-induced diabetic osteopenia in Wistar rats.	Enhanced insulin production, improved bone mass, connectivity, and biomechanical strength; showed synergistic enhancement in the therapeutic effect of hPTH.	[46]
2.	Seed powder (800 mg/kg, p.o.) for 12 weeks	Ovariectomy-induced osteoporosis in female Wistar rats.	NS reversed ovariectomy-induced osteoporosis, possibly via its unsaturated fatty acids, antioxidant, and anti-inflammatory properties.	[99]
3.	Ethanolic seed extract (5, 10, and 20 mg/kg, p.o.) for 8 weeks	Ovariectomy-induced osteoporosis in female Wistar rats.	Treatment with extract enhanced bone matrix thickness and reversed osteoporosis condition through suppression of TRAF6 and NFATc1.	[100]
4.	Seed extract (10 mg/kg, p.o.) for 4 weeks	Ovariectomy-induced calvarial defect model in female Wistar rats.	NS extract treatment enhances bone healing process via enhancing osteoblastic activity.	[10]
5.	Seed powder extract (400, 800, and 1200 mg/kg, p.o.) for 90 days	Ovariectomy-induced osteoporosis in female Wistar rats.	Augmentation in dry bone mass with NS extract treatment, possibly via enhancing osteoblastic activity and reducing osteoclastic activity.	[101]
6.	NS Oil (800 mg/kg, p.o.) for 6 weeks	Steroid-induced osteoporosis in female rats.	Treatment with oil reduced bone resorption and enhanced bone formation via decreased urinary hydroxyproline level and enhanced serum osteocalcin, bone-specific alkaline phosphate, and calcium level.	[104]
7.	Oil (25, 50, and 100 µg)	Osteoblast and myoblast differentiation in C2C12 cell lines.	Enhanced osteoblast and myoblast differentiation.	[105]
8.	TQ (1, 5, 10, 20, 30, and 40 μM)	Bone formation by using MC3T3-E1 osteoblast cells.	TQ potentiated anabolic effects in MC3T3E1 cells via inducing the expression of bone morphogenetic protein-2 and upregulated the phosphorylation of ERK signaling pathway.	[107]
9.	TQ (2.5, 5, 10, 15 μM) TQ (5 mg/kg, p.o.) for 8 days	RANKL-induced osteoclastogenesis in RAW 264.7 and primary bone marrow-derived macrophages cells. LPS-induced bone resorption in male C57/BL6 mice.	TQ suppressed RANKL-induced NF-κB activation via attenuating phosphorylation of IKKα/β, MAPKs, and inhibiting expression of TRAP, DC-STAMP, NFATc1, c-Fos, and ROS level. TQ inhibited bone resorption by suppressing osteoclastogenesis and improving bone mineral density in mice.	[108]
10.	TQ (25, 50, and 100 µM)	Adipose-derived mesenchymal stem cells differentiation.	TQ potentiated stem cell differentiation into the osteoblasts by enhancing collagen, osteopontin level, and osteocalcin gene.	[109]

NS–*Nigella sativa*; TQ–thymoquinone.

**Table 2:** Nanotechnology-mediated drug delivery approach utilized to improve the therapeutic efficacy of *Nigella sativa* and its active constituent (TQ) in different disease conditions.

S. No.	Active Agent	Type of Nanoparticle	Route	Indication	Research Outcome	Ref.
1.	TQ	Cubic phase nanoparticles (cubosomes)	In vitro	Breast Cancer	-Significant improvement in apoptotic activity with TQ-loaded cubosomes in MDA-MB-231 cells. -These cubosomes accumulate in the cytoplasm of MDA-MB-231 cells.	[116]
2.	NS seed extract	Silver nano-composites	In vitro	Hepatocellular carcinoma	-Significant (*p* < 0.05) cytotoxic effect in developed *Nigella sativa* silver nanocomposite system with IC_50_ value 7.16 μg/mL against HepG2 cancer cell.	[117]
3.	NS seed oil	Liposomes	Oral	Antianalgesic	-Oil of *Nigella sativa* seeds containing liposomal formulation exhibited significant analgesic activity in Swiss albino mice.	[118]
4.	TQ	Lipid–polymeric nanoparticles (hybrid NPs)	In vitro	Colon cancer	-TQ-loaded hybrid NPs exhibited improved absorption compared to free TQ in intestinal absorption tests (Caco-2 cells). -TQ-loaded hybrid NPs improved the anticancer activity and minimized the migration of C26 cancer cells more compared to the free TQ.	[119]
5.	TQ	Polymeric NPs	Oral	Pancreatic cancer	-TQ-loaded polymeric NPs exhibited significant anticancer activity against the PANC-1 cancer cell line. -TQ-loaded polymeric NPs exhibited a significant increase in oral bioavailability compared to free TQ in mice. -Investigation signifies its ability to improve the biopharmaceutical characteristics of free TQ.	[120]
6.	NS seed extract	Polymeric micelle	In vitro	Antibacterial	-Antibacterial assay activity reveals a larger zone of inhibition in the case of NS seeds containing polymeric micelle compared to the crude extract.	[121]
7.	TQ	Chitosan-modified solid lipid NPs	Oral	-	-Significant improvement in oral bioavailability of TQ-loaded chitosan-modified solid lipid NPs compared to the TQ suspension.	[122]
8.	TQ-rich fraction	Nanoemulsion	Oral	Alzheimer	-The TQ-rich fraction containing a nanoemulsion system decreased the level of soluble Aβ40 and Aβ42 through modulation of APP processing, ultimately helpful to achieve the anti-Alzheimer activity.	[123]
9.	TQ	Nanoemulsion	Topical	Wound healing	-Significant improvement in the process of healing in the excisional wound in case of TQ containing nanoemulgel compared to the TQ gel	[112]
10.	NS seed extract	NS extract-capped silver NPs	In vitro	Antidiabetic	-A significant antidiabetic activity was observed in the case of NS extract-capped silver nanoparticles in dipeptidyl peptidase IV inhibition assay and α-amylase assay.	[124]

NS: *Nigella sativa*; TQ: thymoquinone; NP: nanoparticles.

## Data Availability

Not applicable.
